# Changes in opioid-related deaths following increased access to opioid substitution treatment

**DOI:** 10.1186/s13011-021-00351-4

**Published:** 2021-02-10

**Authors:** Lisa Andersson, Anders Håkansson, Jonas Berge, Björn Johnson

**Affiliations:** 1 Department of Social Work, Faculty of Health and Society, Malmö University, Malmö, Sweden; 2 Faculty of Medicine, Department of Clinical Sciences Lund, Psychiatry, Lund University, Lund, Sweden; 3 Malmö Addiction Centre, Region Skåne, Malmö, Sweden

**Keywords:** Low threshold, Mortality, Opioid, Opioid-related deaths, Opioid substitution treatment, Sweden, Treatment access

## Abstract

**Background:**

Opioid-related mortality is high and increasing in the Western world, and interventions aimed at reducing opioid-related deaths represent an important area of study. In Skåne County, Sweden, a patient choice reform resulted in increased access to opioid substitution treatment (OST). In addition, a gradual shift towards less restrictive terms for exclusion from OST has been implemented. The aim of this study was to assess the impact of these policy changes on opioid-related deaths.

**Methods:**

Detailed data on opioid-related deaths in Skåne during the 2 years prior to and following the policy change were obtained from forensic records and from health care services. Data on overdose deaths for Skåne and the rest of Sweden were obtained using publicly available national register data. Time periods were used as the predictor for opioid-related deaths in the forensic data. The national level data were used in a natural experiment design in which rates of overdose deaths were compared between Skåne and the rest of Sweden before and after the intervention.

**Results:**

There was no significant difference in the number of deaths in Skåne between the data collection periods (RR: 1.18 95% CI:0.89–1.57, *p*= 0.251). The proportion of deaths among patients enrolled in OST increased between the two periods (2.61, 1.12–6.10, *p*= 0.026). There was no change in deaths related to methadone or buprenorphine in relation to deaths due to the other opioids included in the study (0.92, 0.51–1.63, *p*= 0.764). An analysis of national mortality data showed an annual relative decrease in unintentional drug deaths in Skåne compared to the rest of Sweden following the onset of the reform (0.90, 0.84–0,97, *p*= 0.004).

**Conclusions:**

Opioid-related deaths, as assessed using forensic data, has not changed significantly in Skåne following a change to lower-threshold OST. By contrast, national level data indicate that the policy change has been associated with decreased overdose deaths. The discrepancy between these results highlights the need for more research to elucidate this issue. The result that more patients die during ongoing OST following an increase in access to treatment underlines the need for further preventive interventions within the OST treatment setting.

**Supplementary Information:**

The online version contains supplementary material available at 10.1186/s13011-021-00351-4.

## Background

### Introduction

Opioid-related mortality is high and increasing in the Western world and constitutes a significant public health challenge [1–6]. As in many other Western European countries, opioid-related mortality has increased continously in Sweden over the past two decades [7, 8]*.* It is well established in the research that mortality among opioid-dependent individuals decreases when opioid substitution treatment (OST) is initiated and that mortality is lower for those who stay in such treatment compared to those who discontinue it [9–12]. Less is known about the impact of OST on opioid-related mortality in communities at large, for instance whether increased access to OST reduces such mortality at an aggregate level.

In Sweden, OST has for a long time been conducted with a distinctly rehabilitative ambition and with restrictions on the number of patients receiving treatment. In the case of repeated relapses, treatment has often been terminated through involuntary discharge [13], a practice supported by national regulations. Tendencies towards a more harm reduction-oriented OST have been noted during the 2010s, however, especially in some parts of the country [14, 15]. In parallel with this, both the number of care providers and the number of patients in treatment have increased [16–18].

In the county of Skåne (Scania) in the south of Sweden, there has been a particularly sharp increase in recent years in both the availability of OST and the level of retention in treatment. These changes can partly be attributed to the introduction of a patient choice reform for OST in Skåne in 2014, which led to a marked increase in the number of places in OST in the region, and partly to changes in practice and regulations within OST since the mid-2010s [16, 19].

### High-threshold and low-threshold OST settings

OST can be conducted in many ways, with variations being found both between and within countries. One commonly used distinction is that between programs that diverge in their views on strictness in the treatment setting, known as *high-threshold* and *low-threshold* OST.

High-threshold programs are typically characterized by an idea of rehabilitation, with complete abstinence from illicit drugs (and in some settings alcohol) being required for participation in OST. Exclusion through involuntary discharge follows (repeated) relapses into use or abuse of such substances. Follow-ups of patient compliance in treatment are frequent in the form of supervised on-site drug administration and supervised urine tests. Access to the treatment is often provided through specialist care only. Admission criteria are inflexible and waiting lists are often long due to restrictions on the number of treatment places [20, 21].

Typical low-threshold programs are described as being more harm-reduction oriented, and in contrast to high-threshold programs they do not require abstinence from drug use as a condition of service use. Relapses are expected and strategies to cope with relapses are therefore outlined. Low-threshold programs aim to reduce barriers to service access, such as waiting lists, and advocate more flexible admission criteria, treatment that is free of charge to the patients, and access to OST through both specialist and primary care providers. Low-threshold OST settings are also characterized by less frequent supervision of drug administration and less focus on urine testing as a means of following up patients’ compliance with treatment [20].

### OST in Sweden and Skåne

OST was introduced in Sweden in the late 1960s and has for a long time been implemented within a high-threshold paradigm [14, 15, 22]. Sweden’s restrictive stance on the regulation of OST was traditionally characterized by limitations on the number of patients in treatment, strict admission criteria, and an abstinence-based view on relapse during treatment. In addition to waiting lists to access treatment, a suspension period was applied following involuntary discharge, which prevented patients from seeking treatment again before the specified period had elapsed [17, 23].

Apart from limitations on the length of treatment and costs for medication,^1^ which are often represented in high-threshold OST but do not apply to Swedish programs, OST in Sweden has generally been conducted based on the features that commonly characterize high-threshold and abstinence-based programs.

However, a gradual change has taken place in Swedish OST over recent years towards low-threshold and more harm-reduction oriented OST programs. This change has been particularly evident in Skåne County in southern Sweden [19]. In 2014, the Skåne Regional Council implemented a patient choice reform for OST. The main aims of the reform were to increase access to treatment by permitting private clinics to provide OST, and to strengthen the patients’ influence on their treatment conditions by letting them choose between treatment providers [16]. In parallel with the implementation of the patient choice reform in Skåne, the national guidelines regulating OST were revised in 2016. These changes in the guidelines, towards a more tolerant approach to admission criteria and relapses during OST, were an adjustment to reflect the way OST practice had over time adopted a more lenient approach towards relapse and exclusion, which was particularly evident in Skåne. The suspension period following discharge from treatment was also finally abolished in this revision of the guidelines [24].

The patient choice reform led to a substantial increase in access to OST in Skåne, from 992 to 1453 patients in treatment between 2013 and 2016, an increase of 46%. The number of OST treatment clinics rose from eight to sixteen during the same period. The reform has also resulted in a somewhat larger geographical spread across the region, as the number of municipalities with OST clinics has increased. However, the three largest municipalities, which already had established OST clinics, accounted for 73% of the increase in the number of patients. The largest increase in patients in OST treatment occurred during the initial years of the patient choice reform. The possibility for patients to choose their treatment provider has also led to increased retention rates in OST. Dissatisfied patients can choose to change clinics rather than dropping out of treatment [16, 19].

### Opioid-related deaths and OST

One ambition with the patient choice reform was that increased access to OST would reduce the number of opioid-related deaths in the region. Drug-related mortality in Sweden has increased sharply during the 2000s, and Swedish figures are high by comparison with other European countries [2, 7]. At least 80% of drug-related deaths are opioid related [8]. Since there are no current estimates of the number of people with problematic opioid use, either in Sweden or Skåne, it is not possible to establish the size of the target population.

Low-threshold OST, characterized by rapid and generous access to OST and lower levels of restrictions that may lead to discharge, may result in a higher proportion of opioid-dependent individuals initiating OST and not then being involuntarily excluded from the programs [20, 25]. High participation and retention in OST among the population of opioid-dependent individuals is desirable in order to reduce the risks associated with untreated opioid dependence [11, 20]. As was mentioned above, changes towards more low-threshold treatment have been implemented at a later stage in Sweden than in many other western countries.

However, there are also objections to low-threshold programs. More liberal, harm reduction-based treatment contexts with a lower degree of control may pose a greater risk that patients in OST may divert their medication to drug users who are not in treatment [18, 26–29]. A study on methadone-related deaths in Danish low-threshold OST settings found high prescribed doses of methadone and a high level of prescribed benzodiazepines among the deceased [30]. Such factors may counteract OST’s purpose of preventing opioid-related deaths [11, 31]. Increased access to OST may be associated with both diversion and an increase in opioid-related deaths, as more individuals with problematic opioid use are given access to methadone and buprenorphine, but also with a reduced risk of overdose, if illegal demand decreases when the number of opioid-dependent individuals entering treatment increases [32, 33].

### Objectives

In this study, we investigate opioid-related deaths in Skåne before and after the implementation of the patient choice reform for OST based on regional and national level data. The objective of the study is to examine how the implementation of the patient choice reform may be associated with different aspects of opioid-related mortality. More specifically, we:

1. *Examine the changes in opioid-related deaths in Skåne, and whether the trend differs between areas with increased and unchanged access to OST*. As opioid-related mortality is lower among opioid-dependent individuals who receive OST than among those who use illegal opioids, it is plausible that increased access to OST might lead to a reduced number of opioid-related deaths in Skåne overall. This change would be most noticeable in municipalities in which there has been a marked increase in the number of treatment places.
2. *Examine whether the proportion of deaths in Skåne related to OST medications has changed by comparison with deaths related to other opioids*. Increased access to OST and a more low-threshold, harm reduction-oriented treatment approach may lead to an increased supply of OST medications on the illegal market, partly because a higher number of patients receive treatment, and partly because patients with a high risk of diversion may remain in treatment. At the same time, increased access to OST may lead to reduced demand for illicit OST drugs, as more individuals with problematic opioid use enter treatment.
3. *Examine whether there has been a change in Skåne in the proportion of deceased individuals during ongoing OST*. A transition to more low-threshold oriented treatment would be expected to lead to individuals who had previously risked involuntary discharge instead remaining in treatment. Considering the high risk of intoxication among individuals with problematic use of opioids and other illicit drugs who remain in OST, this would mean that deaths during ongoing treatment may have become more common after the reform (while the number of deaths among involuntarily discharged patients may have decreased).^2^
4. *Examine the development of drug-related deaths in Skåne in relation to the rest of Sweden*. Increased access to OST in Skåne might be expected to lead to lower opioid-related mortality in Skåne in relation to the other regions of the country, in which no corresponding sharp increase in access has taken place, according to national surveys. This analysis serves to control for the general tendency of increasing drug-related mortality that has been noted in Sweden during the last two decades.

## Methods

### Study design and setting

The present study is based on analyses of two different data sets. One of these consists of data on individuals who have died of opioid intoxication during a period of 4 years in Skåne, a county in southern Sweden with approximately 1.4 million inhabitants. The other contains data from the Swedish National Board of Health and Welfare’s (NBHW) national Cause of Death Register. These data sets are hereafter referred to as ‘forensic data’ and ‘national level data’, respectively. Both data sets are described in more detail below. We analyze the data based on the possible impact that the patient choice reform may have had on opioid-related mortality at the regional level.

#### The forensic data set

The forensic data employed in this study were collected in connection with a larger research project, which examined all deceased persons who had been residing in Skåne and who underwent a forensic autopsy during two observation periods of 2 years each, and in whom any opioid had been detected at toxicological analysis. The first period, 1 January 2012 to 31 December 2013, occurred prior to the introduction of the patient choice reform for OST, while the second period, 1 July 2014 to 30 June 2016, took place during the first 2 years of the reform’s implementation.

The data set is based on information from health care and forensic records (including police reports). The data were collected manually via journal review. Data from the forensic and health records were linked at the individual level via the unique personal identification numbers employed in Sweden. The data set also contains information from municipal records and from the Prison and Probation Service, which is not used in this article. We refer to a previous publication from our research group for an extensive and detailed account of authorities and background information [34].

Swedish health care is tax-funded and regionally organized, with a single central organization in Skåne, regardless of whether the care is provided by private or public caregivers. As mentioned previously, OST is only available in Sweden through specialist care provision. Buprenorphine-naloxone is the recommended first-line choice of OST medication [35].

In Sweden, about 90,000 deaths occur annually. Of these, a forensic autopsy is carried out in approximately 6%, including more than 90% of deaths that are drug related [36, 37]. In Skåne, forensic autopsies are conducted at the National Board of Forensic Medicine in Lund on behalf of the police or prosecutor when the cause of death or circumstances surrounding the death are unclear [38, 39]. Forensic medical records contain causes of death, demographic information on the deceased, and the presence and quantity of various substances detected in the toxicological analysis.

Out of a total of approximately 4000 deaths in Skåne for which a forensic autopsy had been conducted during the two periods examined here, opioids were found in 503 deaths. Individuals over 64 years of age (116 cases) have been excluded, since previous Swedish studies have shown that opioid dependence and opioid overdoses are rare among people above the age of 65 [19, 40]. The majority of these deaths were caused by disease, and the presence of the substances of interest to this study were, in most cases, of no significance for the death.

The data set includes individuals for whom the underlying cause of death was considered to be intoxication with heroin, methadone, buprenorphine, fentanyl, oxycodone, or morphine, and where the individuals had a history of illicit drug use. The assessment of whether there was a history of illicit drug use was based on the presence of written information on illicit drug problems in police reports or forensic records, on whether the forensic journals mentioned the presence of injection marks, or if any illegal drug (heroin / 6-Monoacetylmorphine, amphetamine, cocaine or tetrahydrocannabinol (THC)) was found in the forensic toxicological analysis. The reason for assessing whether there was a background of illicit drug use was that we have sought to include individuals who might have met the inclusion criteria for OST, i.e. a diagnosis of opioid dependence. In this way, we have attempted to identify a possible target group for OST, in order to obtain an idea of ​​the impact of OST at the community/county level.

One hundred and ninety-four individuals were further excluded due to the opioids of interest not being present, an absence of information on a history of illicit drug use, or where the cause of death was not considered to be intoxication with any of the opioids of interest. In the majority of these cases, the cause of death was considered to be disease, an accident, or intoxication with substances not examined in this study.^3^

#### The national level data material

One analysis examines drug-related deaths in Skåne by comparison with the rest of Sweden during the years 2011–2017, although excluding the year 2014, during which the patient choice reform was being implemented. The time frame was chosen in order to stay as close as possible to the introduction of the patient choice reform in Skåne, and to avoid interference from other policy changes that may have affected the drug-related mortality in Skåne and Sweden.^4^ For this analysis we use data from the NBHW’s Swedish Cause of Death Register. This register contains annual information on underlying causes of death for nearly all deceased individuals in Sweden, specified using the International Statistical Classification of Diseases and Related Health Problems (ICD) classification [37].

One analysis examines drug-related deaths in Skåne by comparison with the rest of Sweden during the years 2011–2017, although excluding the year 2014, during which the patient choice reform was being implemented. For this analysis we use data from the NBHW’s Swedish Cause of Death Register. This register contains annual information on underlying causes of death for nearly all deceased individuals in Sweden, specified using the International Statistical Classification of Diseases and Related Health Problems.

The NBHW’s Cause of Death Register includes the deaths found in the manually collected forensic data from Skåne. However, the overlap is probably not complete, since the forensic data were not based on diagnostic codes but on the presence of certain opioids, whose (contributory) significance for the death we have then assessed.

The underlying ICD cause of death codes included in the national level data are X42, X44, and Y10–14. This means that the national level data also consists of deaths caused by other opioids than those covered in the forensic data set, as well as poisonings from other drugs (i.e. amphetamines). However, the great majority of overdose deaths in Sweden are opioid-related, and intoxications from the opioids included in the forensic data set make up most of the opioid-related deaths [8]. Population estimates in Skåne and the rest of Sweden are based on census data from Statistics Sweden.

### Measurements and data analysis

Data analysis was performed using IBM SPSS Statistics version 24 for Windows and R version 3.5.3.

#### The forensic data

We divided the cases in the forensic data into two time periods in the form of a dichotomous variable, with the first period referring to the years prior to the patient choice reform (January 2012–December 2013) and the second to the period following the reform (July 2014–June 2016). The reason for using this division, rather than analyzing the trend year by year, is that we have assumed that changes related to the implementation of the intervention should be momentary in nature.

The principal outcome variable in the regression models is the number of opioid intoxication deaths before and after the intervention, adjusted for population size. However, we examine several aspects of these deaths in different analyses. The outcome measures examined are the total number of deaths, deaths during ongoing OST, which substance caused the intoxication (OST drugs versus other opioids), and having a residential address in municipalities with increased or unchanged access to OST following the patient choice reform. Municipalities in which at least one new OST clinic was started during the first year of the patient choice reform have been coded as having increased access to OST. This group includes five municipalities (Malmö, Helsingborg, Lund, Ystad and Landskrona). Of these, OST was already available in the first three prior to the reform. These are also the three largest municipalities in Skåne, comprising 44.8% of the total population in the county. Municipalities in which no new OST clinic was started during the first year of the patient choice reform have been coded as having unchanged access to OST. This group includes 28 municipalities in total. Two of these (Kristianstad and Trelleborg) had OST clinics that were already established prior to the introduction of the patient choice reform. In the remaining 26 municipalities, there was no OST located in the municipality neither before the implementation of the reform nor during the initial year of the patient choice reform. The majority of these 26 municipalities are small, each with a population below 20,000 inhabitants.

Bivariate Poisson regression models have been used to evaluate the relationship between the periods examined and opioid-related deaths for all outcome measures, with the log of the estimated population as offset, yielding rate ratios (RRs) for analysis. Period one was specified as the reference category.

Monthly population estimates (for the 18–65 age range) were obtained from publicly available data from Statistics Sweden [41], and the population size for each period was calculated by averaging the estimates for the period in question (Supplementary Table 1).

Data on the number of patients in OST during the years examined are approximate, since there are no aggregate regional or national statistics on this. The number of patients in treatment was calculated based on data obtained from reports and surveys distributed to treatment providers [19, 42, 43].

#### The national level data

The analysis of national level data used annual numbers of overdose deaths in Skåne County and in the rest of Sweden respectively, and corresponding population estimates (Supplementary Table 2). As described above, data for Skåne County for 2014 were excluded because that was the year in which the intervention started. This analysis employed a natural experiment design, comparing pre- and post-intervention periods for Skåne County by comparison with the rest of Sweden. We employed a model selection process in which several Poisson regression models were compared, each using a different set of independent variables, with the aim of selecting the model with the best fit to the data. Several independent variables were included in the regression models: *year* (assessing the national linear effect of time), *county* (Skåne vs. the rest of Sweden), *intervention level effect* (modeling a shift in mean values after the intervention), and *intervention slope effect* (modeling a shift in the slope of the linear trend after the intervention). Interaction effects between county and intervention level effect (*county X level*), and between county and intervention slope effect (*county X slope*), were also included as variables in the model, and the logarithmized population estimate was used as an offset variable.

Inspecting the graph of overdose deaths visually (see the results section), it was apparent that *year* needed to be included in the model and that there was no baseline effect of *county*. In order to keep the statistical models parsimonious and more easily interpretable, we decided that either *intervention level effect* or *intervention slope effect*, but not both, were eligible for inclusion in the models, and, likewise, either *county X level* or *county X slope*, but not both, were eligible for inclusion. Nine models with all thus eligible combinations of the remaining four variables were computed. The Akaike Information Criterion (AIC) and the Bayesian Information Criterion (BIC) were used for model selection. The best performing model on both information criteria was the model that included year, intervention slope and the interaction effect between county and intervention slope, and this model was therefore chosen as the final model in the present study (Supplementary Table 3).

All of the final Poisson regression models were assessed for overdispersion using the *dispersiontest()* function of the *AER* package in R [44]. None of the models had any statistically significant overdispersion. The Poisson regression models used in the present study are described in an additional file (see Additional file 4).

## Results

### Background of the study population in the forensic data set

One hundred and ninety-three individuals were included in the forensic data set, 88 in the first data collection period and 105 in the second. Descriptive demographic, clinical, and forensic data for the study population are provided in Table 1.

**Table 1:** Characteristics and forensic information for the study population, by data collection period. Numbers (%)

	Period 1 (*n*=88)	Period 2 (*n*=105)	Total (*n*=193)
Age at death, median (Q1;Q3)	33.0 (27.25; 42.75)	35.0 (25.0; 43.0)	34.0 (26.5;43.0)
Male gender	73 (83.0%)	84 (80.0%)	157 (81.3%)
Resident in city with increased access to OST^a^	62 (70.5%)	57 (54.3%)	119 (61.7%)
Stable housing	57 (64.8%)	79 (75.2%)	136 (70.5%)
Opioid substitution treatment, ongoing at time of death or discharged in last year of life	15 (17.0%)	28 (27.2%)	43 (22.5%)
Opioid substitution at time of death	7 (8.0%)	23 (22.3%)	30 (15.7%)
Methadone substitution treatment at time of death	7 (8.0%)	20 (19.0%)	27 (14.0%)
Buprenorphine substitution treatment at time of death	8 (9.1%)	8 (7.6%)	16 (8.3%)
Sedatives (Benzodiazepines, Z-drugs or pregabalin)	79 (89.8%)	83 (79.0%)	162 (83.9%)
Benzodiazepines	67 (76.1%)	73 (69.5%)	140 (72.5%)
CNS stimulants	20 (22.7%)	24 (22.9%)	44 (22.8%)
THC	18 (20.5%)	20 (19.0%)	38 (19.7%)
Alcohol (≥ 0.5‰)^b^	17 (19.3%)	16 (15.2%)	33 (17.1%)
Heroin intoxication ^c^	19 (21.6%)	21 (20.0%)	40 (20.7%)
Methadone intoxication ^c,d^	41 (46.6%)	43 (41.0%)	84 (43.5%)
Buprenorphine intoxication ^c^	12 (13.6%)	18 (17.1%)	30 (15.5%)
Fentanyl intoxication ^c,e,f^	11 (12.5%)	17 (16.2%)	28 (14.5%)
Oxycodone/morphine intoxication ^c,g^	5 (5.7%)	6 (5.7%)	11 (5.7%)

Period 1: 1 January 2012 to 31 December 2013; Period 2: 1 July 2014 to 30 June 2016

^a^Malmö, Helsingborg, Lund, Landskrona and Ystad

^b^Two cases were removed since the analyses were not carried out on femoral blood

^c^In most deaths, the intoxication included more than one substance (often opioids and benzodiazepines). The table shows the number of cases in which specific opioids had caused or essentially contributed to the death

^d^Including two cases in which the fatal intoxication was caused by the ingestion of both methadone and buprenorphine

^e^Including seven cases in which fentanyl findings involved acrylfentanyl, acetylfentanyl or furanylfentanyl (all in data collection period 2)

^f^Fentanyl is in Sweden prescribed in the form of patches, tablets or nasal spray

^g^Of these, eight were oxycodone-related and three were morphine-related (all morphine cases were in data collection period 1)

### Opioid-related deaths in Skåne and in areas with increased or unchanged access to OST

**Fig. 1:**
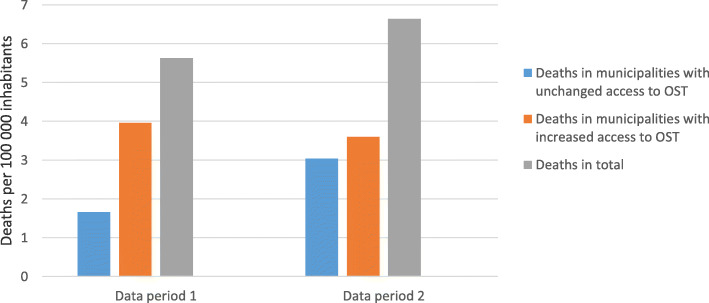
Opiod-related deaths in Skåne in total and grouped by municipality of residence, by period

The analyses of the forensic material from Skåne indicate that there was an increase in the number of deaths due to intoxication with heroin, methadone, buprenorphine, fentanyl, oxycodone, or morphine in the county between the two data collection periods; from 88 during period 1 to 105 during period 2. When adjusted for population estimates, the increase was not significant (RR = 1.18 [0.89–1.57], *p* = 0.251) (Fig. 1).

The analysis of the effect of the patient choice reform on deaths in municipalities with increased or unchanged access to OST respectively, showed a significant increase in the proportion of deaths in municipalities with unchanged access between period 1 and period 2 (RR=1.85 [1.15–2.98], *p* = 0.011). The effect of the intervention on the proportion of deaths registered in municipalities with increased access to OST between the data collection periods was non-significant (RR=0.90 [0.63–1.29], *p* = 0.561). An analysis of the interaction effect showed a significant difference in the change from period 1 to period 2 with regard to the proportion of deceased persons who were resident in municipalities with increased access to OST at the time of death, as compared with those who were resident in municipalities with unchanged access to OST. This means that the proportion of the deceased who were resident in municipalities with increased access to OST decreased by comparison with municipalities with unchanged access to OST following the implementation of the reform (RR=0.49 [0.27–0.88], *p* = 0.018).

### Deaths related to OST medications or other opioids

**Fig. 2:**
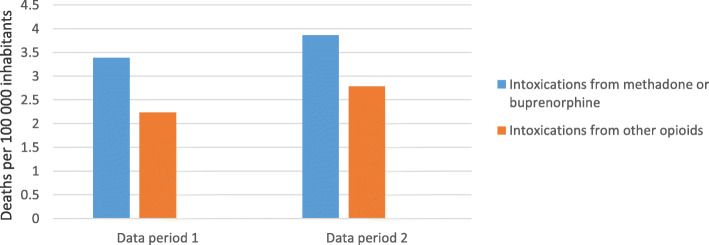
Opioid-related deaths in Skåne grouped by the substances that caused the intoxication, by period

The proportion of Skåne’s population aged 18–65 who died from intoxication with the OST medications methadone or buprenorphine did not change significantly between the data collection periods (RR = 1.14 [0.79–1.65], *p* = 0.489). Nor was any significant change between the periods observed in the analyses of deaths due to intoxication with other opioids (RR = 1.24 [0.80–1.94], *p* = 0.335), or in the analysis of deaths from intoxication with OST medications, viewed in relation to the proportion who died as a consequence of intoxication with heroin, fentanyl, oxycodone, or morphine (RR = 0.92 [0.51–1.63], *p* = 0.764) (Fig. 2).

### Deaths during ongoing OST

The study has also analyzed changes between the observation periods in the proportion of individuals who died from intoxication caused by the opioids included in the study during ongoing OST in Skåne viewed in relation to the number of treatment places in OST. The results show that the proportion of deaths during ongoing OST increased by 161% from period 1 to period 2 (RR=2.61 [1.12–6.10], *p* = 0.026).

### Drug-related deaths in Skåne and the rest of Sweden

**Fig. 3:**
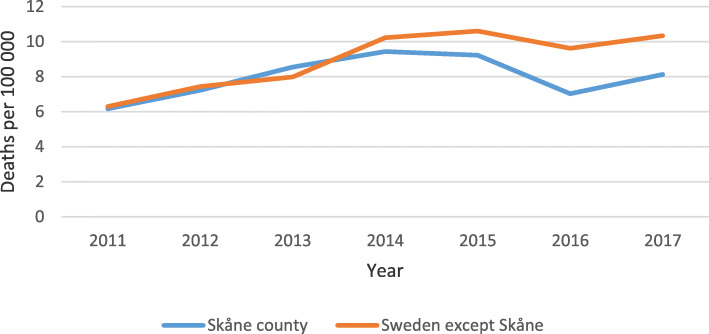
Drug-related deaths in Skåne and the rest of Sweden 2011–2017

Finally, we analyzed national mortality data from the NBHW regarding the proportion of deceased individuals aged 20–64 who died from unintentional drug intoxication in Skåne compared with the rest of Sweden for the period 2011–2017 (Fig. 3). The final model included *year*, *intervention slope effect*, and *county X intervention*. The main effect of chronological year was an increase of 17% per year (RR = 1.17, 95% CI: 1.13–1.22, *p* < 0.001) and the national level change in slope after the intervention was minus 14% per year (RR = 0.86, 95% CI: 0.80–0.91, p < 0.001). The main effect of interest to the present study, *county X intervention* (i.e. the difference in the post-intervention slope change between Skåne and the rest of Sweden) was a decrease of 10% per year (RR = 0.90, 95% CI: 0.84–0.97, *p* = 0.004).

## Discussion

In this study, we have examined drug-related deaths in the southern Swedish county of Skåne as the level of access to OST has increased sharply following the introduction of a patient choice reform in 2014, in parallel with a gradual shift towards increased symptom tolerance and a more harm reduction-oriented approach in OST treatment. In summary, OST in Skåne is now conducted more in accordance with a low-threshold paradigm than was previously the case [16, 19].

One aim of the implementation of the patient choice reform was that deaths due to opioids would decrease in the region as a result of increased access to OST for individuals in need of such treatment [19, 45]. OST provision has previously been under-dimensioned both in the region and in the rest of the country [46, 47]. In absolute numbers, there has been an increase in deaths in Skåne due to intoxication with the opioids examined in the study, although analyses of the number of deaths in the region adjusted for population estimates show no significant increase in the mortality rate between the data collection periods. However, the analysis based on national data from the NBHW’s Cause of Death Register for 2011–2017 showed a reduction in unintentional drug intoxications in Skåne after 2014 relative to the rest of the country. The differences in these results can be attributed to the two analyses’ different inclusion criteria for the study populations of interest (with regard to substances and causes of death), different time periods (the forensic material from Skåne includes 2 years that do not run per calendar year), and the fact that the data from the NBHW cover more years. However, taken together, the results from these analyses suggest that there has not been a significant increase in opioid-related deaths in Skåne following the implementation of the patient choice reform. At the same time, support for the expectation that the reform would be associated with a reduction in opioid deaths in Skåne is weak.

The proportion of individuals who at the time of death were registered as resident in municipalities with unchanged access to OST increased between the data collection periods. The expansion in the number of patients receiving OST may have helped to restrain opioid-related mortality in municipalities with improved access to OST, where opioid-dependent individuals are able to initiate OST more easily and may now also remain in treatment regardless of relapses. A previous evaluation has shown proximity to the clinic to be important for patients in OST when choosing a treatment facility [19]. In recent years, there has been a shift in several western countries towards an increasing number of opioid deaths in less densely populated communities outside metropolitan areas [48–50]. In this study, the three larger municipalities in Skåne were the places in which access to OST had increased the most and no increase in the proportion of deaths was noted in these. Municipalities with unchanged access to OST, on the other hand, are largely comprised of areas with longer geographical distances to an OST clinic. This group mainly consists of a large number of smaller municipalities. It is possible that the results in the present study in a way reflect this shift towards opioid use and deaths being more common in smaller communities. In this context, however, it should be clarified that a shortcoming in the current analysis is the premise that proximity to treatment, i.e. that OST is available in the municipality, is desirable in order to reach the aim of a reduction in opioid-related deaths. At the same time, it is unrealistic for most of the smaller communities in Skåne to have an OST-clinic located in each municipality.

Prior to the introduction of the patient choice reform, a concern was raised that an expansion of the number of patients in treatment, and thus an increase in individuals receiving medication in the form of methadone and buprenorphine, could lead to increased diversion of these medications to people not in OST. This is something that has been observed in other studies where access to OST has increased [17, 18, 27, 30, 51, 52]. The analyses in this study show that there was no significant increase in deaths caused by methadone or buprenorphine in general in Skåne between periods examined. There has been a change in which opioids cause the most deaths in many western countries since the turn of the millennium, from heroin to prescription opioids, including methadone and buprenorphine [53–56]. This trend has also been noted in Sweden [8, 40, 57]. In a recently published study Fugelstad and colleagues showed that methadone-related deaths have increased among Swedish 15–29-year olds between 2006 and 2015. Only a few individuals had been prescribed methadone the year before the death, which indicates that the increasing trend was mainly a result of an increased exposure to non-prescribed methadone among younger opioid users [57]. Another recent study by Mariottini and colleagues [58] investigated buprenorphine-related deaths in Finland 2016–2019. The results show high concomitant use of primarily benzodiazepines in buprenorphine poisonings and indicate an increase in buprenorphine-related deaths, especially among younger individuals. The study did not examine the proportion among the deceased who had prescriptions of buprenorphine or experience of OST [58]. The results of these, and other, studies illustrate a current increase in deaths related to OST-medications. In contrast to this, the increased prescription of methadone and buprenorphine linked to the patient choice reform has, in the present study, not been associated with increased regional mortality as a result of intoxication with these substances.

When patients, as in this study, are able to initiate and remain in OST even though they may have a potentially life-threatening use of other narcotic drugs, the fact that a larger proportion of patients in the present study died during ongoing treatment in the second data collection period might be viewed as a natural consequence of this. One possible explanation for this finding, in addition to the large increase in the number of patients in treatment, may be that OST-patients are not being discharged from treatment to the same extent as before the introduction of the patient choice reform, in combination with the changes in national guidelines, as presented in a previous study [19]. In line with this, a recent study from low-threshold OST programs in Norway found that more than half of the deaths during OST over 2 years were drug-induced [59]. The authors express concern about the large number of cases where an opioid, including the patient’s prescribed OST medication, was the main intoxicant in overdose deaths. Higher pooled concentrations of opioids were found among the drug-induced deaths compared to those who died from other causes. Among the deceased in overdose who were prescribed buprenorphine, other opioids (mainly heroin) were found to a high extent [59]. In our study, we have not investigated the concentration of substances at death, nor do we know which opioid caused the deaths in the individuals who were on OST at time of death. Nevertheless, the findings in these studies highlight the aspect of caution related to patients with concomitant use of non-prescribed drugs in more low-threshold OST settings. It should also be noted that the ability of the OST clinics to reach patients with risky opioid use for preventive measures such as naloxone distribution and overdose information may be greater than before, with more patients with lower treatment compliance and an ongoing use of illicit or prohibited drugs remaining in treatment [17, 34, 60].

### Strengths and limitations

Possible strengths of the present study include the fact that the forensic data cover a complete regional population of individuals subject to forensic examination and that the manual searches reduce the risk of missing cases that should be included in the study. The unique personal identification number provides easy linkage between patient registers. The national level data from the NBHW’s Cause of Death Register are of high quality in terms of their level of completeness with regard to the inclusion of the total number of deaths that occur in Sweden annually.

One of the study’s limitations is that we lack data for the prevalence and development of illicit use of opioids and opioid dependence in Skåne and Sweden during the years covered by the study.

The number of opioid-related deaths examined forensically has increased continuously in Sweden in the last two decades. Concern has been raised if this increase to a substantial degree could be attributed to improvements and changes in forensic toxicology [61]. This has been thoroughly analyzed by Leifman [8], who concludes that the increase in the presence of drugs in forensically investigated deaths mainly is due to an increase in the actual number of opioid-related deaths, even when taking into consideration factors such as lowered threshold values in forensic toxicology analyses and an increasing screening for drugs. Almost all changes in toxicological analyses and screening took place before 2012, which means that they cannot have affected our regional level data. However, it cannot be ruled out that the changes to a minor extent may have affected the national level data.

Another limitation of the study concerns the fact that both data sets consist of relatively few cases, which diminishes the possibility of drawing general conclusions regarding opioid-related deaths on the basis of the study’s results. Another weakness is the shortness of the time that elapsed between the two data collection periods, and that a gradual change towards a more tolerant attitude towards relapse and the exclusion criteria employed in OST practice had begun prior to and continued during the period in which the empirical data were collected. However, the major impact of the examined intervention, i.e. a sharp increase in the number of treatment places and patients in OST, can be attributed to the implementation of the patient choice reform [19]. The choice of study design by division into time periods rather than analyzing year by year was made in order to best address the research questions concerning the effects of the introduction of the reform on opioid deaths in the region.

## Conclusion

Increased access to and lower treatment thresholds in OST have not led to a reduction in opioid-induced deaths in the short term in Skåne County in southern Sweden. There has been an increase in the actual number of such deaths in the county, but this increase was not significant when adjusted for population size. An analysis of national death data, however, shows a relative decrease in unintentional drug intoxication deaths in Skåne compared to the rest of Sweden following the implementation of the patient choice reform for OST in Skåne. This discrepancy between the results from these different analyses calls for more research to further elucidate this matter.

Deaths due to intoxication with the OST drugs methadone and buprenorphine showed no increase between the data collected prior to and after the introduction of the patient choice reform, indicating that the hypothesis that a higher number of patients in OST might lead to increased mortality due to the diversion of such medications was not supported.

A geographical comparison between individuals who at the time of death were registered as resident in municipalities with increased or unchanged access to OST showed an increase in the proportion of deaths in municipalities with unchanged access to OST.

Following the increase in access to and in the number of patients in OST in Skåne, a larger proportion of patients died during ongoing OST following the introduction of the reform. This can probably be attributed to the greater number of OST clinics and the increase in the number of patients in treatment, and to the policy change which has meant that non-compliance with treatment, in terms of illicit drug use during treatment, no longer necessarily leads to discharge. One implication of the results relating to the increase in deaths among individuals receiving OST at the time of death is the significance of the way in which OST staff work with patients with an ongoing use of illicit or prohibited drugs*.* OST should therefore be highlighted as an important area for overdose prevention measures aimed at this group of problematic opioid users, such as naloxone distribution and information on safer drug use.

## Supplementary Information

**Additional file 1: Supplementary Table 1**. Population aged 18–65 in Skåne in total and in communities with increased or unchanged access to OST in the second data period.

**Additional file 2: Supplementary Table 2**. Population aged 20–64 in Skåne, Sweden except Skåne, and total in Sweden in 2012–2017.

**Additional file 3: Supplementary Table 3**. Model selection for analyses of national level data.

**Additional file 4.** Poisson regression models used in the study.

## Data Availability

The SPSS data set that includes the 193 forensically examined opioid-related fatalities generated and analyzed during the current study is not publicly available due to restrictions made by the Regional Ethical Review Board in Lund, Sweden, but is available from the corresponding author upon reasonable request. The data obtained from the National Board of Health and Welfare are available in the NBHW’s cause of death database. https://sdb.socialstyrelsen.se/if_dor/val_eng.aspx

## Abbreviations

AIC: Akaike Information Criterion
BIC: Bayesian Information Criterion
ICD: The International Statistical Classification of Diseases and Related Health Problems
NBHW: The Swedish National Board of Health and Welfare
OST: Opioid substitution treatment
RR: Rate ratio
SPSS: Statistical package for the social sciences
THC: Tetrahydrocannabinol

## Footnotes

1 Medication costs in Sweden are restricted to a maximum of SEK 2300 (≈ € 230) per patient and year.

2 The proportion of deaths among patients involuntarily discharged from OST cannot be examined as we lack information on the total number of individuals who were involuntarily discharged prior to and after the patient choice reform.

3 There were only 15 individuals < 65 years of age in the larger material that included all opioid deaths in Skåne who died as a result of intoxication with heroin, methadone, buprenorphine, fentanyl, morphine or oxycodone, who were excluded from the study due to lack of a history of illicit drug use. In nine of these cases the manner of death was suicide. Only five individuals were below 40 years of age.

4 Data for 2018 and 2019 have been excluded from the comparison, mainly because of two important national policy changes that occurred in early 2018: (1) Naloxone programs were introduced in several regions, including Stockholm and Skåne, which may have affected drug mortality. (2) Two internet-based sellers of fentanyl analogues were prosecuted for involuntary manslaughter in February 2018, which led to the effect that the online supply of these substances practically ceased overnight. The number of deaths related to fentanyl analogues decreased by 90% between 2017 and 2018, from 101 cases in 2017 to 11 cases in 2018.
