# Supplementary material for: Changes in opioid-related deaths following increased access to opioid substitution treatment
Source: Subst Abuse Treat Prev Policy. 2021 Feb 10;16:15. doi: 10.1186/s13011-021-00351-4 (PMC7876792; doi:10.1186/s13011-021-00351-4)
Supplement: Supplementary file 1 — Additional file 1: Supplementary Table 1. Population aged 18–65 in Skåne in total and in communities with increased or unchanged access to OST in the second data period. [file 13011_2021_351_MOESM1_ESM.docx]

Supplementary Table 1. Population aged 18-65 in Skåne in total and in communities with increased or unchanged access to OST in the second data period

| \| Year/Dates \| Data period \| Skåne \| Communities with increased access to OST \| Communities with unchanged access to OST \|  \| \| --- \| --- \| --- \| --- \| --- \| --- \| \| 2012 \| 1 \| 781 448 \| 401 448 \| 380 000 \|  \| \| 2013 \| 1 \| 783 699 \| 404 980 \| 378 689 \|  \| \| 1 July 2014-  30 June 2015 \| 2 \| 788 792 \| 410 781 \| 378 001 \|  \| \| 1 July 2015-  30 June 2016 \| 2 \| 792 958 \| 414 006 \| 378 952 \|  \| |
| --- | --- | --- | --- | --- | --- | --- | --- | --- | --- | --- | --- | --- | --- | --- | --- | --- | --- | --- | --- | --- | --- | --- | --- | --- | --- | --- | --- | --- | --- | --- |
| Population was estimated as the sum of the number of inhabitants per month in the investigated time periods / 12  Population data was retrieved from Statistics Sweden |
