# Supplementary material for: Changes in opioid-related deaths following increased access to opioid substitution treatment
Source: Subst Abuse Treat Prev Policy. 2021 Feb 10;16:15. doi: 10.1186/s13011-021-00351-4 (PMC7876792; doi:10.1186/s13011-021-00351-4)
Supplement: Supplementary file 2 — Additional file 2: Supplementary Table 2. Population aged 20–64 in Skåne, Sweden except Skåne, and total in Sweden in 2012–2017. [file 13011_2021_351_MOESM2_ESM.docx]

Supplementary Table 2. Population aged 20-64 in Skåne, Sweden except Skåne, and total in Sweden in 2012-2017

| Year | Skåne | Sweden except Skåne | Sweden |
| --- | --- | --- | --- |
| 2011 | 730 284 | 4 777 866 | 5 508 150 |
| 2012 | 733 960 | 4 802 373 | 5 536 332 |
| 2013 | 737 778 | 4 829 248 | 5 567 026 |
| 2014 | 742 592 | 4 861 474 | 5 604 066 |
| 2015 | 748 130 | 4 896 558 | 5 644 688 |
| 2016 | 755 102 | 4 940 831 | 5 695 932 |
| 2017 | 763 402 | 4 987 733 | 5 751 136 |

Population was estimated as (the number of inhabitants on December 31 the present year + the number of inhabitants on December 31 the year before) /2

Population data was retrieved from Statistics Sweden
