# Supplementary material for: Changes in opioid-related deaths following increased access to opioid substitution treatment
Source: Subst Abuse Treat Prev Policy. 2021 Feb 10;16:15. doi: 10.1186/s13011-021-00351-4 (PMC7876792; doi:10.1186/s13011-021-00351-4)
Supplement: Supplementary file 3 — Additional file 3: Supplementary Table 3. Model selection for analyses of national level data. [file 13011_2021_351_MOESM3_ESM.docx]

Supplementary Table 3. Model selection for analyses of national level data

| *Model* | *Variables* | *df* | *AIC* | *BIC* |
| --- | --- | --- | --- | --- |
| *model 1* | year | 2 | 134.6 | 135.8 |
| *model 2* | year + intervention mean | 3 | 134.3 | 136.0 |
| *model 3* | year + intervention slope | 3 | 112.2 | 113.9 |
| *model 4* | year + county X mean | 3 | 126.7 | 128.4 |
| *model 5* | year + county X slope | 3 | 124.2 | 125.9 |
| *model 6* | year + intervention mean + county X mean | 4 | 127.4 | 129.6 |
| *model 7* | year + intervention mean + county X slope | 4 | 124.4 | 126.6 |
| *model 8* | year + intervention slope + county X mean | 4 | 105.7 | 108.0 |
| *model 9* | year + intervention slope + county X slope | 4 | 105.3 | 107.6 |
