# Supplementary material for: Changes in opioid-related deaths following increased access to opioid substitution treatment
Source: Subst Abuse Treat Prev Policy. 2021 Feb 10;16:15. doi: 10.1186/s13011-021-00351-4 (PMC7876792; doi:10.1186/s13011-021-00351-4)
Supplement: Supplementary file 4 — Additional file 4. Poisson regression models used in the study. [file 13011_2021_351_MOESM4_ESM.docx]

**Poisson regression models used in the study**

The Poisson regression model is a generalized linear model in which the conditional mean of the dependent variable is assumed to follow a Poisson distribution, using a log link function. The basic equation for the Poisson regression can be expressed as follows:

$$\log(Y)={}_{0}+{}_{1}X_{1}$$

In which Y represents the dependent variable, X1 to Xn represents the list of independent variables, β0 represents the estimated intercept and β1 to βn represents the estimated parameters in the model corresponding to the independent variables.

In a standard Poisson regression, the dependent variable is modeled as a count variable; i.e. a discrete variable taking any non-negative integer values. However, a rate can be modeled using an offset variable:

$$\log(\frac{Y}{P})={}_{0}+{}_{1}X_{1}$$

Where P in this case represents the total population to which the count of Y refers, so that what is modeled is the fraction of the population that experiences the event of Y. Next, the equation can be rewritten:

$$\log(Y)={\log P+}_{0}+{}_{1}X_{1}$$

The population variable P is now represented in the equation as an offset variable; i.e. there is no parameter estimate for the variable, and it enables the modelling of a rate rather than a count.

Below follow the equations for all models used in the study:

Model 1 (skriv vilken analys som avses och vilken sida I manuset den finns på):

$$\log(Y)={\log P+}_{0}+{}_{1}X_{1}$$

In which Y is the total number of opioid intoxications per one-year interval, P is the total population in Skåne County per one-year interval, and X is a dichotomous variable taking the value 0 for the periods prior to the OST reform and 1 to the periods following the reform.

Model 2:

$$\log(Y)={\log P+}_{0}+{}_{1}X_{1}$$

This model is similar to model 1, but restricted so that Y refers only to the number of opioid intoxications in communities with no increased access to OST and, likewise, P refers to the total population of the same communities.

Model 3:

$$\log(Y)={\log P+}_{0}+{}_{1}X_{1}$$

This model is the same as model 2, but now restricted so that Y and P instead refers to communities with increased access to OST.

Model 4:

$$\log(Y)={\log P+}_{0}+{}_{1}X_{1}+{}_{2}X_{2}+{}_{3}{X_{1}*X}_{2}$$

This is a more complex model with an interaction effect. Y represents the total number of opioid intoxication deaths in communities with or without increased OST access, respectively. Likewise, P represents the population in communities in Skåne with or without increased OST access, respectively. X1 refers to the time period as in the previous models, and X2 refers to a dichotomous variable in which aggregate data from communities with no increased OST access take the value 0 and data from communities with increased access to OST take the value 1. X1 * X2 is the interaction between period and OST access, and B3 is interpreted as the interaction effect, i.e. the difference in proportion of deaths in the two kinds of communities prior to and following the OST reform.

Model 5:

$$\log(Y)={\log P+}_{0}+{}_{1}X_{1}+{}_{2}X_{2}+{}_{3}{X_{1}*X}_{2}$$

Similar to model 4, this is a more complex model with an interaction effect. Y represent opioid intoxication deaths, both by methadone/buprenorphine and by other drugs, and P represents the total population in Skåne County for each time interval. X1 refers to the time period as in the previous models, and X2 refers to a dichotomous variable in which observations of methadone/buprenorphine death counts take the value 1, and observations of deaths counts by other drugs take the value 0. The interaction effect B3 is interpreted as the change in the proportion between methadone/buprenorphine deaths and other drug deaths following the OST reform.

Model 6:

$$\log(Y)={\log P+}_{0}+{}_{1}X_{1}$$

This model is similar to previous models in which P represents the total population of Skåne County for each time interval and X represents the periods prior to and following the intervention. In this model, Y is limited to deaths by methadone or buprenorphine intoxications only.

Model 7:

$$\log(Y)={\log P+}_{0}+{}_{1}X_{1}$$

This model is similar to model 6, but Y is limited to deaths by other opioids than methadone or buprenorphine.

Model 8:

$$\log(Y)={\log P+}_{0}+{}_{1}X_{1}$$

This model is structurally similar to previous models, but with some important differences. In this model, Y is restricted to opioid intoxication deaths occurring in patients in ongoing OST. Likewise, P is the estimated number of patients in active OST in Skåne County for each time interval in order to estimate the change of mortality rate within the OST programs in Skåne County following the OST reform.

Model 9:

$$\log(Y)={\log P+}_{0}+{}_{1}X_{1}+{}_{2}X_{2}$$

This is a more complex model based on the national level data. Y represents the number of opioid related deaths for each time interval, per County group (Skåne County vs. rest of Sweden in one group). Likewise, P refers to the total population per County group for each time interval. X1 refers the chronological years of 2011-2017. X2 refers to an interaction variable between year, Skåne County, and OST reform, which means that the interpretation of B2 is the yearly (multiplicative) difference between Skåne County and the rest of Sweden following the intervention. The data table is as follows:

| County group | Year | Opioid deaths | Population | County X reform |
| --- | --- | --- | --- | --- |
| Skåne | 2011 | 45 | 730284 | 0 |
| Skåne | 2012 | 53 | 733960 | 0 |
| Skåne | 2013 | 63 | 737778 | 0 |
| Skåne | 2014 | - | - |  |
| Skåne | 2015 | 69 | 748130 | 1 |
| Skåne | 2016 | 53 | 755102 | 2 |
| Skåne | 2017 | 62 | 763402 | 3 |
| Rest of Sweden | 2011 | 301 | 4777866 | 0 |
| Rest of Sweden | 2012 | 357 | 4802373 | 0 |
| Rest of Sweden | 2013 | 386 | 4829248 | 0 |
| Rest of Sweden | 2014 | 497 | 4861474 | 0 |
| Rest of Sweden | 2015 | 519 | 4896558 | 0 |
| Rest of Sweden | 2016 | 475 | 4940831 | 0 |
| Rest of Sweden | 2017 | 515 | 4987733 | 0 |

Note that the variable *County group* is not actually used in the final model, as it was dropped during the model selection procedure. Because 2014 was the year in which the OST reform took place, the data from Skåne County from this year was excluded since it might be considered a transition period.
